# Association of preoperative cerebrospinal fluid sTREM2 concentration with postoperative delirium and 3-year mortality in patients total knee or hip arthroplasty: a prospective cohort study

**DOI:** 10.1097/JS9.0000000000003465

**Published:** 2025-09-10

**Authors:** Bin Wang, Xiao Wang, Yizhi Liang, Jiahan Wang, Shuhui Hua, Jian Kong, Shanling Xu, Yunchao Yang, Yanan Lin, Chuan Li, Hongyan Gong, Xu Lin, Yanlin Bi

**Affiliations:** a Department of Anesthesiology, Qingdao Municipal Hospital, Qingdao, Shandong Province, China; b Department of Anesthesiology, Binzhou Medical University, Binzhou, Shandong Province, China; c Department of Anesthesiology, Shandong Second Medical University, Weifang, Shandong Province, China

**Keywords:** anesthesiology, cerebrospinal fluid, cohort study, delirium, neuropsychological tests, sTREM2, surgery

## Abstract

**Background:**

As a common postoperative neurological complication, postoperative delirium (POD) can lead to poor postoperative recovery in patients, prolonged hospitalization, and even increased mortality. However, POD’s mechanism remains undefined and there are no reliable molecular markers of POD to date. The present work examined the associations of cerebrospinal fluid (CSF) soluble triggering receptor expressed on myeloid cells 2 (sTREM2) with CSF POD biomarkers, and investigated whether the effects of CSF sTREM2 on POD were modulated by the core pathological indexes of POD (Aβ_42_, tau, and ptau). The association of presurgical CSF sTREM2 with 3-year mortality in POD cases administered total knee or hip arthroplasty was assessed.

**Methods:**

We enrolled 545 Chinese Han patients undergoing total knee or hip arthroplasty (aged 50–95 years, weighing 50–80 kg, and using ASA II–III) combined with epidural anesthesia between October 2020 and March 2022. In these participants, POD was identified using the Confusion Assessment Method (CAM) and the severity of POD was evaluated using the Memorial Delirium Assessment Scale (MDAS) at 1–7 days postoperatively (or before discharge) by an anesthesiologist. The levels of CSF POD biomarkers were measured by ELISA. Next, logistic regression models were used to analyze the association between sTREM2and POD, as well as between cerebrospinal fluid (CSF) biomarkers and POD. We used Stata MP16.0. to examine whether the association between sTREM2 and POD was mediated by CSF POD biomarkers. We also used potential predictive factors to built 5 models, including Logistic Regression (LR), Support Vector Machine (SVM), K Nearest Neighbours (KNN), AdaBoost and CatBoost to assess the predictive abilities of sTREM2. After that, we verified the performance of the 5 models in the set, plotting receiver operating characteristic (ROC) curve analysis and precision recall curve (PRC) were used to further evaluate whether the machine learning (ML) models were effective in supporting clinical decision-making. All POD patients were followed up for 3 years, and Kaplan–Meier (K–M) survival analysis was used to compare the 3-year mortality rates of high sTREM2 group and low sTREM2 group in patients with POD.

**Results:**

Finally, a total of 545 patients (122patients in POD group and 423in NPOD group) were included in our study. sTREM2 and CSF levels of tau and ptau in the POD group were higher than those in the NPOD group. CSF Aβ_42_, Aβ_42_/ tau, and Aβ_42_/ ptau in the POD group were lower than those in the NPOD group. CSF sTREM2 was negatively associated with Aβ_42_ (*r* = −0.445, *P* < 0.001), Aβ_42_/ tau (*r* = −0.350, *P* < 0.001) and Aβ_42_/ ptau (*r* = −0.429, *P* < 0.001), CSF sTREM2 was positively associated with tau (*r* = 0.179, *P* = 0.048) and ptau (*r* = 0.311, *P* < 0.001). The relationship between sTREM2 and POD was partially mediated by tau and ptau, with the mediation proportion of 17.91% and 22.09%, respectively. The following five variables (sTREM2, age, tau, ptau, and Aβ42/ptau) were significant predictive factors via Lasso regression. Meanwhile, univariable analysis demonstrated CSF Aβ_42_/ptau levels was the protective factor of POD and sTREM2, age, tau, ptau were the risk factors of POD. Upon adjusting for possible confounders, including education level, sex, MMSE score, as well as history of diabetes, smoking, drinking, and hypertension, multivariable analysis showed consistent results. Following two rounds of sensitivity analysis, our results remained robust.The ROC(AUC = 0.999, 95% CI:0.999–1.000) and PRC(AUC = 0.998, 95% CI: 0.995–1.000) for CatBoost were significantly better than the other models. The dynamic online calculator can accurately predict the occurrence of POD by selecting POD patients for the internal validation study. The Kaplan–Meier curve showed no significant difference in survival probability between the low sTREM2 group and high sTREM2 group (log-rank *P* = 0.53), but age subgroup analysis revealed significantly between age≥80 plus sTREM2 ≥ 20 000 pg/ml subgroup and the other subgroups on mortality in patients with POD (log-rank *P* = 0.017).

**Conclusion:**

Elevated CSF sTREM2 is a preoperative risk factor for POD, which is partially mediated by tau and ptau. The CatBoost model can accurately predict the occurrence of POD. Age≥80 plus sTREM2 ≥ 20 000 pg/ml could increase 3-year mortality in POD cases.

## Introduction

Postoperative delirium (POD) is a serious complication of anesthesia and surgery^[1]^. POD features transient or permanent cognitive impairment, deteriorated language comprehension, and reduced social adaptation. It induces further complications, including Alzheimer’s disease (AD), long-lasting hospitalization, elevated treatment expenses^[2]^, and even elevated mortality. Glumac showed that preoperative corticosteroid administration ameliorates inflammatory response induced by surgery, and thereby reduced the incidence and severity of cognitive complications^[3]^. However, POD’s mechanism remains undefined and there are no reliable molecular markers of POD to date.

Amyloid beta (Aβ), which encompasses Aβ_40_ and Aβ_42_, represents the key constituent of senile plaques in AD. Tau, a neuronal protein, is important in microtubule assembly and stabilit^[4]^. Recent evidence shows preoperative positive cerebrospinal fluid (CSF) Aβ and Tau elevate the odds of delirium post-surgery^[5]^. Further data revealed preoperative positive CSF Aβ, total tau (tau), and phosphorylated tau (ptau) independently predict postsurgical delirium upon elective arthroplasty in elderly cases not previously diagnosed with dementia. Aβ and tau are important factors in POD pathogenesis^[6]^.

TREM2 belongs to the immunoglobulin family, a cell surface receptor that is selectively highly expressed on microglia in the central nervous system and regulates phagocytosis, cytokine production, cell proliferation, and cell survival^[7]^. TREM2 undergoes proteolytic cleavage and is released into the extracellular space as soluble triggering receptor expressed on myeloid cells 2 (sTREM2), which is selectively highly expressed on microglia^[8]^, regulates phagocytosis, cytokine production, cell proliferation, and cell survival. sTREM2 can be detected in cerebrospinal fluid, plasma, and serum. These changes in sTREM2 concentrations in body fluids may be potential cues to microglial dysfunction and neuroinflammation typical of neurodegeneration^[9]^. sTREM2 can inhibit the apoptosis of giant cells and promote their proliferation and migration, protecting existing neuron cells from damage^[10]^. Studies have shown that sTREM2 levels in the cerebrospinal fluid of Alzheimer’s patients are elevated and peak in the early stages of the disease^[11]^. Since POD and AD have comparable neuropathological mechanisms, we hypothesized that CSF sTREM2 is associated with POD.

**HIGHLIGHTS**

- Elevated CSF sTREM2 is a preoperative risk factor for POD, which is partially mediated by tau and ptau.
- The CatBoost model can accurately predict the occurrence of POD.
- Age ≥80 plus sTREM2 ≥ 20 000 pg/ml could increase 3-year mortality in POD cases.

We hypothesized that there is a significant association between CSF sTREM2 and POD and that CSF biomarkers mediate this relationship. In addition, we hypothesized that CSF sTREM2 would have effects onthree-year mortality in patients with POD.

This cohort/cross-sectional/case-control study was reported per the STROCSS guidelines.

## Methods

### The PNDABLE study

Investigating perioperative pathologies linked to neurocognition, PNDABLE aimed to assess the related pathogenetic mechanisms, risk elements, and molecular markers, focusing on the identification of PND lifestyle risk factors in Han individuals of northern China. PNDABLE may help generate standardized models for timely prevention and diagnosis of the identified conditions in non-demented Han individuals. Prior to presurgical CSF and blood collection, each participant provided signed informed consent. This study, entered in the Chinese Clinical Trial Registry, was approved by the Ethics Committee of Hospital. PNDABLE belongs to a prospective cohort study.

### Participants

PNDABLE recruited Han Chinese participants aged 50–95 years, with a weight range of 50–80 kg, and ASA physical status II or III. All participants underwent total knee or hip arthroplasty under epidural anesthesia from October 2020 to March 2022. The study employed stringent exclusion criteria to ensure a homogenous participant pool. These criteria included: (1) Preoperative Mini-Mental State Examination (MMSE) score below 24 points; (2) a history of substance abuse, e.g., administration of psychotropic medications or prolonged use of steroids or hormonal medications; (3) presurgical stage III or IV hepatic encephalopathy; (4) recent history of complex surgical procedures; (5) significant visual or auditory impairments that could hinder participation in neuropsychological testing; (6) preoperative evidence of abnormal coagulation that could increase surgical risk; (7) preexisting neurological conditions such as multiple sclerosis, head injury, central nervous system infections, neurodegenerative diseases (excluding AD) like epilepsy or Parkinson’s disease, or other major neurological diseases; (8) diagnosis of significant psychiatric illnesses; (9) critical systemic diseases (e.g., malignant cancer) that could potentially alter CSF or blood levels of AD biomarkers, including amyloid-beta (Aβ) and tau protein; (10) family history of genetic disorders.

**Figure 1.**
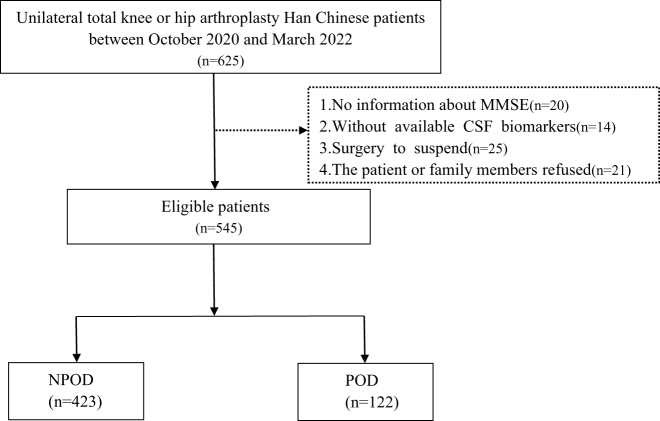
The flow diagram showed the selection of eligible patients and the enrollment process. POD: postoperative delirium; NPOD: no postoperative delirium.

The PNDABLE study enrolled 625 participants with normal cognitive function who provided data on various relevant variables. The participants were subsequently categorized into two groups considering the postoperative cognitive dysfunction (POD) status, i.e., the POD and non-POD (NPOD) groups. Figure 1 shows a detailed study flowchart. Preoperatively, the patients were specifically instructed to abstain from food for 8 h and fluids for 6 h before their scheduled surgery.

### Anesthesia and surgery

Upon arrival in the operating theater, the patients were administered a lumbar anesthesia. A lumbar puncture was performed at the L3–L4 interspace. Following successful needle placement, 2 mL of CSF was aspirated from the subarachnoid space. Subsequently, 0.66% ropivacaine (approximately 2 to 2.5 mL) was administered by intrathecal injection over a duration approximating 30 s. To maintain hemodynamic stability during surgery, participants received supplemental oxygen via face mask at 5 L/min. The treatment aimed to keep blood pressure within ± 20% of baseline values. In case noninvasive blood pressure (NIBP) decreased to <90 mmHg or fell by more than 20% from baseline, bolus ephedrine at 5 mg was intravenously injected. Likewise, when heart rate (HR) decreased to <50 beats per minute (bpm), atropine at 0.5 mg was intravenously administered. To conduct postsurgical pain management, a patient-controlled intravenous analgesia (PCIA) system was implemented following surgery. The PCA regimen consisted of butorphanol (0.1 mg/mL) mixed with tropisetron (50 µg/mL) in 100 mL normal saline. The VAS score was maintained at less than 3 points after the operation for 3 days. Following surgery, participants underwent transfer to the post-anesthesia care unit (PACU) for continual monitoring and management.

In the preoperative period, all participants underwent comprehensive baseline data collection 24 h prior to surgery. These data included demographic information such as age, gender, body mass index (BMI), and education level. Additionally, pertinent medical history details were thoroughly retrieved from participants’ medical records. These details encompassed smoking status (defined as current smokers who consumed at least one cigarette daily for one year or former smokers who had quit), alcohol consumption history (defined as drinking at least once a week for more than one year), and past diagnoses of hypertension (reflected by blood pressure ≥140/90 mmHg) and diabetes mellitus (defined as fasting venous blood glucose ≥7.0 mmol/L, random intravenous blood glucose ≥11.1 mmol/L, or 2-h blood glucose ≥11.1 mmol/L following the glucose tolerance test). A qualified anesthesiologist was responsible for collecting medical history, performing physicals, and administering cognitive tests for dementia.

### CSF POD biomarkers and CSF sTREM2 assessments

CSF specimens obtained as described above were processed within a 2-h timeframe to ensure optimal preservation. Each sample underwent centrifugation at 2000 × g for 10 min. Subsequently, CSF samples were aliquoted and stored in enzyme-free Eppendorf (EP) tubes (AXYGEN; PCR-02-C) at −80°C. The latter procedures were implemented in accordance with the international BIOMARKAPD project for facilitating their utilization in downstream analyses. Quantification of CSF markers associated with POD and CSF sTREM2 was performed with enzyme-linked immunosorbent assays (ELISAs) utilizing a Multiskan MK3 plate reader (Thermo Scientific). The employed ELISA kits targeted Aβ_42_ (BioVendor, Belgium; No. 296-64401), tau (BioVendor, No. EK-H12242), ptau (BioVendor, No. QY-PF9092), and sTREM2 (Human TREM2 SimpleStep ELISA kit; Abcam, No. Ab224881). ELISAs were conducted as directed by the respective manufacturers by experienced technicians in a blinded manner. For minimizing inter-assay variability, duplicate measurements were performed for various samples and standards, and mean values were subsequently analyzed. Furthermore, to ensure result reliability, all antibodies and plates utilized belonged to the same lot. The within-batch coefficient of variation (CV) was <5% (mean CV 4.5% for Aβ_42_, 2.5% for ptau, and 4.4% for tau). The inter-batch CV was <20% (mean CV 5.3% for Aβ_42_, 2.4% for ptau, and 4.8% for tau).

### Neuropsychological tests

Preoperatively, all participants were submitted to a cognitive test employing the Mini-Mental State Examination (MMSE) scale administered by a neurologist. Individuals with MMSE scores lower than 24 points were excluded to ensure baseline cognitive normalcy.

POD assessment was conducted twice daily by an anesthesiologist from 9:00-10:00 AM to 2:00-3:00 PM, commencing on postoperative day 1 and continuing until postoperative day 7 or hospital discharge. Concurrent with delirium assessment, pain intensity was evaluated with a visual analog scale (VAS) that ranged between 0 (no pain) and 10 (worst pain imaginable). Diagnosis of POD was established by the Confusion Assessment Method (CAM)^[12]^, while POD severity was evaluated with the Memorial Delirium Assessment Scale (MDAS)^[13]^. Patients with positive test results were assigned to the POD group, with the remaining ones categorized into the non-POD (NPOD) group.

### Statistical analysis

Based on preliminary analysis, seven covariates were identified for potential inclusion in the logistic regression model. For ensuring sufficient statistical power, a sample size of 625 participants was calculated, assuming a 20% loss-to-follow-up rate and a POD incidence of 10%. Data normality for continuous variates was evaluated by the Kolmogorov–Smirnov test, and those with normal and skewed distributions were presented as mean ± standard deviation (SD) and median (Q25, Q75) or percentage, respectively. For group comparisons, the chi-square test was used for categorical variables, while the Mann–Whitney U-test was employed for continuous variables, based on data distribution. The application of the above statistical tests was strategically made considering the inherent characteristics of the data to optimize the analysis of group disparities.

The associations of CSF sTREM2 with CSF biomarkers of POD (Aβ_42_, tau, ptau, Aβ_42_/tau, and Aβ_42_/ptau) were also examined by multivariable linear regression after adjustment for age, sex, and education level.

We further investigated the potential moderating effect of CSF POD markers on the association of sTREM2 with POD using Stata MP16.0 (StataCorp LLC, USA). Specifically, we employed a three-equation, mediator–moderator model framework with logistic regression. In the first equation, CSF POD biomarkers (mediator) were regressed on sTREM2 (independent variable). The second equation examined the independent effect of sTREM2 on POD (dependent variable). Finally, the third equation included both sTREM2 and CSF POD biomarkers as independent variables to assess their combined effect on POD. To quantify the potential attenuation or indirect effect of CSF POD biomarkers, bootstrapping was performed with 10 000 iterations, controlling for age, education level, gender, and MMSE score in various models.

Variable selection was conducted via the least absolute shrinkage and selection operator (LASSO) method.Independent variables with nonzero coefficients in the LASSO regression model were selected and subsequently analyzed via multivariate logistic regression(*P* < 0.05) to identify potential predictive factors.Logistic regression analysis was conducted to investigate whether potential predictive factors independently affect POD and whether they are risk factors for or protective factors of POD in SPSS (version 23.0). To enhance the transparency and interpretability of variable selection, a directed acyclic graph (DAG) was constructed. All candidate variables for the multivariate model were entered simultaneously using the Enter method to control for potential covariances between variables. To reduce the risk of potential multicollinearity, a variance inflation factor (VIF) analysis was performed to examine the potential shared variance among the variables.

We use potential predictive factors to built five models, including Logistic Regression (LR), Support Vector Machine (SVM), K Nearest Neighbours (KNN), AdaBoost, and CatBoost via R project software (version 4.3.2). The selection of model hyperparameters used 10× cross-validation on the training dataset. Cross-validation ensured a better evaluation of model performance by averaging the measurements across multiple trials. After that, we verified the performance of the five models in the set, plotting receiver operating characteristic (ROC) curve analysis and precision recall curve (PRC) were used to further evaluate whether the ML models were effective in supporting clinical decision-making. Furthermore, calibration was employed to validate the predictive model’s accuracy. Finally, a nomogram including the determined independent variables was developed to facilitate further analysis. Ten POD cases examined between April 2022 and April 2023 were chosen for internal validation.

Overall and nonlinear relationships between continuous sTREM2 and POD were examined by restricted cubic spline analysis (RCS), with the reference set to the optimal cutoff. Then, patients were categorized by sTREM2 levels per the optimal cutoff, and Kaplan–Meier curve analysis was conducted for assessing survival differences. Next, multivariable Cox proportional hazards models were employed to examine the association of sTREM2 with mortality in patients with POD who were followed up for three years via R project software (version 4.3.2).

Statistical significance was set at *P* ≤ 0.050.

### Statement

The work has been reported in line with the STROCSS criteria^[14]^.

## Results

### Comparing the characteristics and CSF biomarker levels of participants

**Figure 2.**
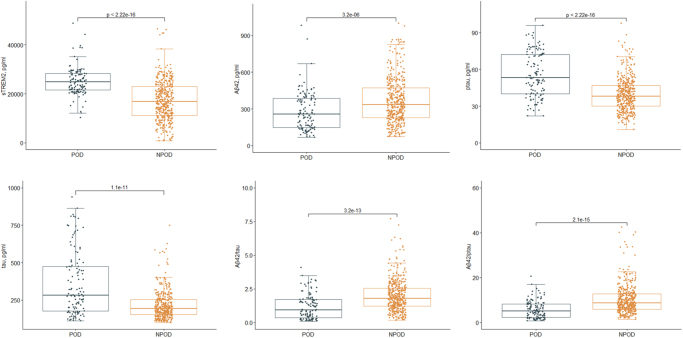
Distribution of CSF sTREM2 and biomarkers levels for participants with and without POD during subsequent hospitalization by Mann–Whitney U-test for non-normal. POD: postoperative delirium; NPOD: no postoperative delirium.

POD incidence was 28.8% (122/423). The POD and NPOD groups were significantly different (*P* < 0.05) in sTREM2 and multiple CSF markers, e.g., Aβ_42_, tau, ptau, Aβ_42_/tau, and Aβ_42_/ptau, as shown in Figure 2. Interestingly, both groups were similar in gender, BMI, education level, MMSE score, smoking or drinking history, hypertension, diabetes, surgery duration, estimated volume of infusion, estimated blood loss, and anesthesia duration. Patients’ clinicodemographic data are summarized in Table 1.

**Table 1:** Characteristics of participants in PNDABLE

Characteristic	NPOD (*n* = 423)	POD (*n* = 122)	*P*
Age [year, M(IQR)]	59 (11)	74 (8)	0.000
Male [*n* (%)]	259 (61.2)	63 (51.6)	0.058
BMI [kg.m^−2^, M(IQR)]	25.39 (5.06)	25.49 (4.88)	0.770
Education [year, M(IQR)]	9 (6)	9 (6)	0.782
MMSE [scores, M(IQR)]	28 (3)	28 (2)	0.220
Smoking history [*n* (%)]	123 (29.1)	28 (23.0)	0.183
Drinking history [*n* (%)]	103 (24.3)	21 (17.2)	0.098
Hypertension [*n* (%)]	182 (43.0)	63 (51.6)	0.092
Diabetes [*n* (%)]	82 (19.4)	31 (25.4)	0.148
MDAS [M (IQR)]	1 (6)	13 (5)	0.000
sTREM2 [pg/ml,M (IQR)]	16 906.79 (11 873.03)	24 863.68 (6762.07)	0.000
Aβ_42_ [pg/ml, M (IQR)]	335.34 (241.86)	258.72 (243.17)	0.000
ptau [pg/ml, M (IQR)]	37.96 (17.02)	53.17 (32.06)	0.000
tau [pg/ml, M (IQR)]	188.56 (103.56)	279.96 (303.46)	0.000
Aβ_42_/ptau [M (IQR)]	8.78 (6.87)	5.07 (6.08)	0.000
Aβ_42_/tau [M (IQR)]	1.78 (1.35)	0.93 (0.82)	0.000
Duration of anesthesia [min, M (IQR)]	140 (30)	140 (40)	0.060
Duration of surgery[min, M (IQR)]	120 (30)	120 (30)	0.375
Estimated volume of infusion [ml, M (IQR)]	900 (200)	900 (200)	0.233
Estimated blood loss [ml, M (IQR)]	110 (80)	110 (50)	0.905

Continuous variable use Student’s *t*-test or Mann–Whitney U, Categorical variable use chi-square test.

The numerical variables of normal distribution are statistically described by Average ± Standard deviation [x̅ ± s].

Non-normally distributed numerical variables are statistically described by Median (Interquartile spacing) [M (Q)].

Categorical variables are statistically described by sample size (percent) [*n* (%)].

Abbreviations: POD: postoperative delirium; NPOD: no postoperative delirium; M: median; IQR:interquartile range;

BMI: body mass index; MMSE: minimum mental state examination; MDAS: Memorial Delirium Assessment Scale.

The bold values are all *P* ≤ 0.05, statistically significant parameters.

### Associations of CSF sTREM2 and CSF markers of postsurgical delirium

**Figure 3.**
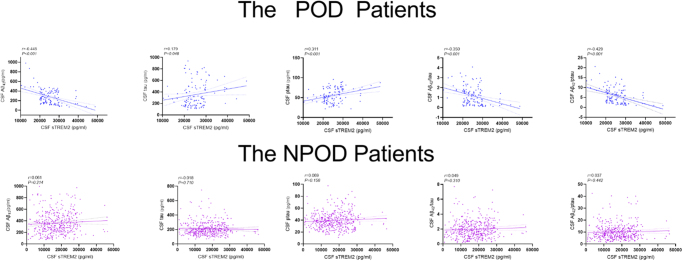
Correlation analysis between CSF sTREM2 and POD in patients. Scatter plots represent the associations of CSF sTREM2 with CSF biomarkers (Aβ_42_, tau, ptau, Aβ_42_/tau, and Aβ_42_/ ptau) of POD. The normalized regression coefficients (*r*) and *P*-values computed by multiple linear regression after adjustment for age, sex, and educational level. POD: postoperative delirium; NPOD: no postoperative delirium.

The associations of CSF sTREM2 with CSF markers of POD were examined in linear regression models, with adjustment for age, sex, and education level. As shown in Figure 3, CSF sTREM2 had negative associations with Aβ_42_ (*r* = −0.445, *P* < 0.001), Aβ_42_/tau (*r* = −0.350, *P* < 0.001), and Aβ_42_/ptau (*r* = −0.429, *P* < 0.001), and positive associations with tau (*r* = 0.179, *P* = 0.048) and ptau (*r* = 0.311, *P* < 0.001) in POD cases. However, CSF sTREM2 levels were not significantly correlated with Aβ_42_ (*r* = 0.061, *P* = 0.214), Aβ_42_/tau (*r* = 0.049, *P* = 0.310), Aβ_42_/ptau (*r* = 0.037, *P* = 0.442), tau (*r* = .018, *P* = 0.710), and ptau (*r* = 0.069, *P* = 0.156) in NPOD cases. The above data suggested elevated CSF sTREM2 was associated with lower Aβ_42_ content and higher levels of tau pathology (Fig. 3).

### Causal mediation analyses

**Figure 4.**
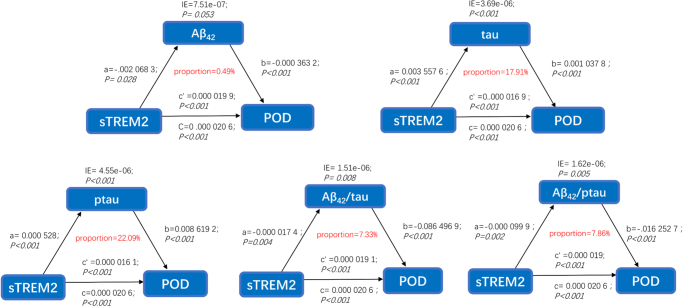
Mediation analyses with 10 000 bootstrapped iterations were used to examine the mediation effects of Aβ_42_, tau, ptau, Aβ_42_/tau, and Aβ_42_/ ptau on POD. POD: postoperative delirium.

The association between sTREM2 and POD was partially mediated by tau and ptau, with mediation proportions of 17.91% and 22.09%, respectively (Fig. 4A-E).

### Lasso regression analysis and logistic regression analysis of factors potentially affecting POD

To investigate the risk and protective factors for POD, we first constructed a directed acyclic graph (DAG) to enhance the transparency of variable selection. The results of the DAG analysis are presented in Supplemental Digital Content, Figure S1 available at: http://links.lww.com/JS9/F107. In the collinearity analysis, the VIF values of the confounding factors were all less than 10, indicating no collinearity among these confounding factors; the specific results are shown in Supplemental Digital Content, Table S1 available at: http://links.lww.com/JS9/F106.

**Figure 5.**
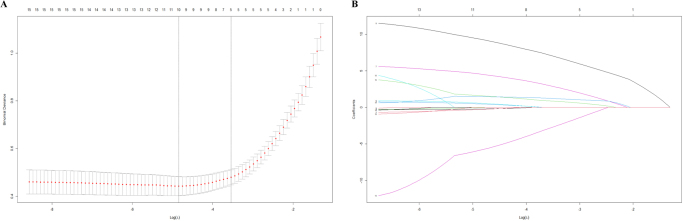
(A) Binomial deviance vs. Log(λ). (B) Coefficient paths for different variables. (A) Illustration of the relationship between binomial deviance and Log(λ). Each red dot represents the binomial deviance for a specific λ value, with error bars denoting the standard error of the deviance. The plot demonstrates that as Log(λ) increases, the binomial deviance increases and determines the optimal λ. (B)depicts the coefficient paths for various variables, with the *x*-axis representing Log(λ) and the *y*-axis representing the coefficients of the variables. Different colored lines denote different variables. As Log(λ) changes, the coefficients of most variables approach zero. Ultimately, five variables with nonzero coefficients were identified, underscoring their importance in the model.

Among the 15 potential predictors, the following five variables (sTREM2, age, tau, ptau, and Aβ_42_/ptau) were significant predictive factors via Lasso regression and SHapley Additive exPlanations (SHAP) in Figure 5 and Supplemental Digital Content, Figure S2 available at: http://links.lww.com/JS9/F108.

We then conducted univariate logistic regression analyses by including sTREM2, age, tau, ptau, and Aβ_42_/ptau. The five variables had significant independent associations with POD. Meanwhile, univariable analysis demonstrated CSF Aβ_42_/ptau levels was the protective factor of POD and sTREM2, age, tau, ptau were the risk factors of POD. Upon adjusting for possible confounders, including education level, sex, MMSE score, as well as history of diabetes, smoking, drinking, and hypertension, multivariable analysis showed consistent results. Following two rounds of sensitivity analysis, our results remained robust, with CSF Aβ_42_/ptau levels continuing to show statistical significance. Moreover, as demonstrated, sTREM2, age, tau, and ptau remained significant risk factors for POD in Table 2.

**Table 2:** Logistic regression on analysis and sensitivity analysis in PNDABLE study

	Model 1^a^		Model 2^b^		Model 3^c^	
	OR (95% CI)	*P*	OR (95% CI)	*P*	OR (95% CI)	*P*
sTREM2, pg/ml	1.000 (1.000–1.000)	0.000	1.000 (1.000–1.000)	0.000	1.000 (1.000–1.000)	0.000
age, year	1.335 (1.265–1.409)	0.000	1.337 (1.266–1.412)	0.000	1.346(1.272–1.424)	0.000
ptau, pg/ml	1.065 (1.050–1.080)	0.000	1.065 (1.049–1.080)	0.000	1.067 (1.052–1.083)	0.000
tau, pg/ml	1.007 (1.005–1.008)	0.000	1.007 (1.005–1.008)	0.000	1.007 (1.005–1.008)	0.000
Aβ_42_/ptau	0.808 (0.761–0.859)	0.000	0.809 (0.761–0.860)	0.000	0.803 (0.755–0.854)	0.000

OR = odds ratio; 95%CI = 95% confidence interval; A**β**_42_ = **β**-amyloid42; T-tau = total tau; P-tau = phosphorylated tau.

^a^ Model 1: Unadjusted.

^b^ Model 2: Adjusted for sex, years of education, and MMSE scores.

^c^ Model 3: Adjusted for sex, years of education, MMSE scores, smoking history, drinking history, hypertension, and diabetes

The bold values are all *P* ≤ 0.05, statistically significant parameters.

### Model development and internal validation in POD cases

Each machine learning model has its own unique approach when dealing with classification problems. Therefore, different models have their respective advantages and disadvantages when making predictions. Due to the complex pathological mechanism of POD disease and the incomplete understanding of the interactions among risk factors, we chose five machine learning models in order to utilize our rich dataset to determine which model can predict POD most accurately.

**Figure 6.**
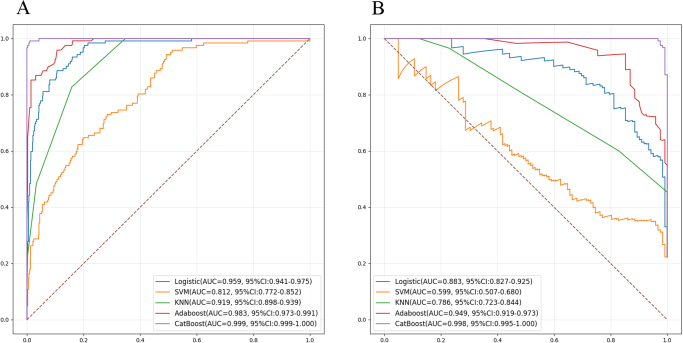
Model AUC–ROC curves and Model AUC–PRC curves. (A) Receiver operating characteristic curves for five POD prediction models. (B) Precision recall curves for five POD prediction models.

In ROC curve analysis, the AUC–ROC was 0.959 (95% CI 0.941–0.975) for Logistic. The AUC–ROC was 0.812 (95% CI 0.772–0.852) for SVM. The AUC–ROC was 0.919 (95% CI 0.898–0.939) for KNN. The AUC–ROC was 0.983 (95% CI 0.973–0.991) for Adaboost. The AUC–ROC was 0.999 (95% CI 0.999–1.000) for CatBoost. Similarly, PRC curve analysis supported these findings. The AUC-PRC was 0.883 (95% CI 0.827–0.925) for Logistic. The AUC– PRC was 0.599 (95% CI 0.507–0.680) for SVM. The AUC–PRC was 0.786 (95% CI 0.723–0.844) for KNN. The AUC–PRC was 0.949 (95% CI 0.919–0.973) for Adaboost. The AUC–PRC was 0.998 (95% CI 0.995–1.000) for CatBoost (Fig. 6A-B).

**Figure 7.**
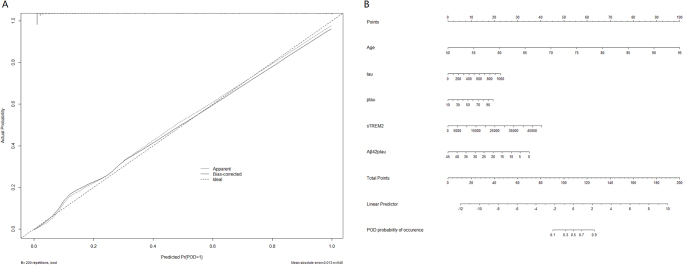
(A) Calibration curve presented prediction of POD after surgery between the prediction model and actual observation. The Hosmer–Lemeshow test indicated a good prediction of the nomogram. (B) Nomogram for predicting POD after surgery.

**Figure 8.**
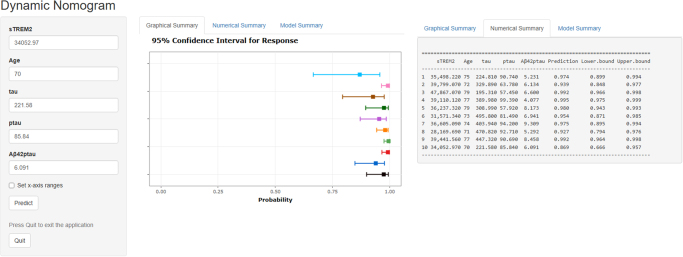
The online web-based calculator for predicting POD. The online calculator translated from the nomogram for generating risk of POD by the internal verification. Users can submit values for the seven features into the corresponding text box of the web page through the computer or mobile phone for calculation. Once the output of the sample has been calculated, the results page will display the probability of POD, the 95% confidence interval, and the parameters of the model.

A calibration plot comparatively assessing model-predicted and actual POD rates was designed based on the Hosmer–Lemeshow test (*P* = 0.551), suggesting good predictive accuracy (Fig. 7A). A nomogram including the determined independent variates was developed (Fig. 7B) and converted into a dynamic online calculator (https://thedile.shinyapps.io/dynnomapp/) to promote the clinical application of the current data. The dynamic online calculator could accurately predict POD through selection of POD cases in the internal validation study (Fig. 8).

### Association of sTREM2 and mortality in patients with POD

**Figure 9.**
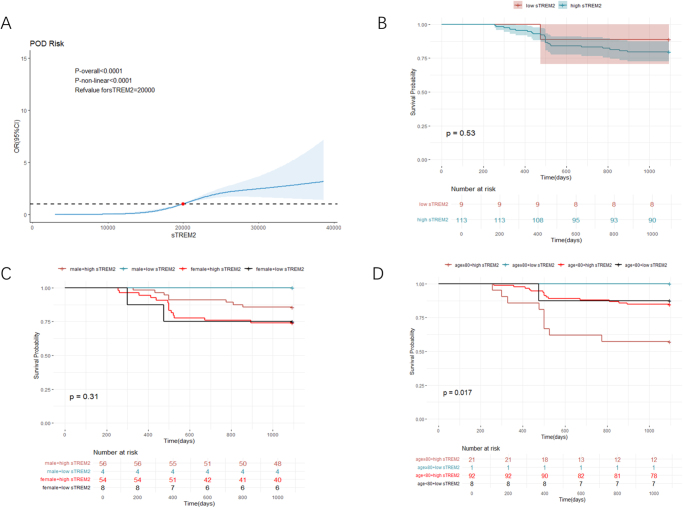
Restricted cubic spline analysis and Kaplan–Meier curves for 3-year mortality in the POD patients.

The study followed up 122 patients with POD for 3 years, of whom 98 patients survived and 24 died, for an overall survival rate of 19.67%. RCS analysis was employed to demonstrate the correlation between sTREM2 and POD in patients (*P*-overall < 0.0001), but this association was not non-linear (*P*-non-linear = 0.2636). The original model revealed a “S-shaped” relationship, indicating that patients with higher sTREM2 faced an increased risk of POD with sTREM2 greater than 20 000 pg/ml (Fig. 9A). Kaplan–Meier curve analysis demonstrated similar survival odds in the low-sTREM2 and high-sTREM2 groups (log-rank *P* = 0.53) (Fig. 9B). Furthermore, subgroup analysis based on sex showed sTREM2 did not significantly interact with sex to affect mortality in patients with POD (log-rank *P* = 0.31) (Fig. 9C). But subgroup analysis based on age demonstrated significant interaction between sTREM2 and age to affect mortality in patients with POD (log-rank *P* = 0.017) (Fig. 9D). The results of multivariate Cox proportional hazards models revealed that age≥80 plus sTREM2 ≥ 20 000 pg/ml carried the highest risk of mortality in patients with POD.

## Discussion

In this study, POD incidence was 28.8%, corroborating the reported POD incidence ranging between 3.6% and 41%^[15]^. CSF sTREM2 and CSF Aβ_42_, tau, ptau, Aβ_42_/tau, and Aβ_42_/ptau levels had significant differences between the POD and NPOD groups. The study showed CSF sTREM2 independently predicted POD. Furthermore, this risk factor may be associated with specific CSF biomarkers(tau and ptau). Among the 15 potential predictors, the following 5 variables (sTREM2, age, tau, ptau, and Aβ_42_/ptau) were significant predictive factors via Lasso regression and SHAP. Few previously published investigations evaluated the association of sTREM2 and mortality in patients with POD. The above findings showed age≥80 plus sTREM2 ≥ 20 000 pg/ml could increase 3-year mortality in the POD patients. Therefore, these results suggest that early intervention to reduce sTREM2 can improve long-term outcomes.

Aβ is crucial for the progression of many neurological disorders, including AD. Specifically, Aβ aggregation and accumulation show tight correlations with AD development and aggravation^[16]^. Injection of condensed Aβ into the cerebral cortex in rat and monkey models causes cortical tissue necrosis, gliosis, and peripheral neuronal loss, with significant and positive concentration-dependent correlations with the administered Aβ, as determined via electron microscopy^[17]^. High hippocampal Aβ content postanesthesia is a potential mechanism of POCD. Recent evidence suggests cerebral Aβ content has a causal association with cognitive impairment^[18]^. Additionally, direct injection of condensed Aβ into the rat hippocampus decreases learning and memory^[19]^. Tau protein, detected in central nervous cells in great amounts, stabilizes microtubules. Abnormal phosphorylation, glycosylation, and ubiquitination of tau protein functionally impairs nerve fibers^[20]^. A report demonstrated Aβ biosynthesis and tau phosphorylation participate in AD development^[21]^. Intracranial administration of agglutinated Aβ induced tau phosphorylation at specific sites and neuron apoptosis. The hippocampus is the brain region most involved in learning and memory, especially short-term memory^[22]^. Deposited Aβ is very neurotoxic, particularly targeting hippocampal cells. Evidence indicates hippocampal administration of Aβ markedly reduces learning and memory capabilities in rats, and the receptor for advanced glycation end-products (RAGE) in microglial cells partly mediates the neurotoxic effects of Aβ. Aβ interacts with RAGE and activates microglia to release abundant cytotoxic compounds, including inflammatory mediators and free radicals, causing apoptotic death^[23]^. Enhanced biosynthesis, altered metabolism, and impeded transport of Aβ may induce its accumulation in the brain, resulting in severe neurotoxicity.

TREM2 is a transmembrane protein that binds ligands through the outer domain, activates microglia and promotes phagocytosis by activating intercellular signaling pathways^[24]^. In addition, cell culture and animal experiments have its extracellular domain is cleaved by various abscisic enzymes to produce soluble TREM2 (sTREM2), which has an independent function from TREM2 and regulates the interaction between neurons and the microenvironment^[25]^. sTREM2 is produced by alternative splicing of transcripts, enters the extracellular space and regulates a variety of pathophysiological processe^[26]^. Most current studies on sTREM2 have found that its activation in vivo can inhibit the inflammatory response, especially in microglia and macrophages. Activation of sTREM2 can inhibit the intracellular PI3K/AKT pathway and NF-κB activation^[27]^, suppress the TLR4 pathway, and reduce the production of related pro-inflammatory cytokines, e.g., IL-6 and TNF-alpha. On the other hand, sTREM2 promotes the differentiation of microglia or macrophages from pro-inflammatory M1 cells to anti-inflammatory M2 cells. Thus, the central or peripheral inflammatory response is weakened^[28]^. The myelogenic macrophages of sTREM2 knockout mice were activated by TLR ligands, and the production of interferon (IFN) and TNF-alpha increased significantly^[29,30]^. Overexpression of sTREM2 on the surface of microglia could lead to a reduction of TNF-alpha production^[31]^. In addition, sTREM2 can also regulate the phagocytic function of microglia and improve the deposition of Aβ in the brain^[32]^. In 2013, a report pointed out genetic variants of sTREM2 participate in the pathological process of Alzheimer’s disease, and researchers found a rare missense mutation in the sTREM2 gene (rs75932628-T) that replaces the original R47H site, resulting in a significantly increased risk of AD. At the same time, it was found that 80 to 100-year-old people without dementia who carry the rs75932628-T gene mutation have worse cognitive function compared with non-carriers^[33]^. These studies all suggest that sTREM2 is crucial for maintaining the function and cognitive ability of microglia. Previous studies have found that CSF sTREM2 level can predict neurofibrillary degeneration and cognitive impairment and has high diagnostic value in differentiating between diseased and healthy individuals^[34]^. Therefore, this study chose to observe cerebrospinal fluid sTREM2.

This study found that higher CSF sTREM2 was independently and strongly associated with the CSF levels of POD biomarkers. CSF sTREM2 had negative associations with Aβ_42_, Aβ_42_/tau and Aβ_42_/ptau and positive correlations with tau and ptau in POD cases. In addition, we also demonstrated that CSF POD biomarkers (CSF tau and ptau) exert a mediating effect between CSF sTREM2 and POD.

Under normal physiological conditions, site-specific phosphorylation of tau protein at specific epitopes regulates its interaction with microtubules. However, excessive phosphorylation of tau has been shown to impair neuronal structure and function. Hyperphosphorylated tau exhibits reduced capacity to bind microtubules and promote microtubule assembly, leading to intracellular accumulation. This accumulation disrupts axonal transport, suppresses acetylcholine release, and inhibits proteasome activity, collectively contributing to slow progressive neurodegeneration. The dissociation of hyperphosphorylated tau from microtubules facilitates its polymerization into paired helical filaments (PHFs) that eventually generate neurofibrillary tangles (NFTs). Glycogen synthase kinase-3β (GSK-3β), one of the two primary kinases responsible for tau phosphorylation, regulates glycogen synthesis by inactivating glycogen synthase and phosphorylating target proteins. Additionally, GSK-3β participates in extracellular signaling pathways governing energy metabolism and neuronal development. Elevated soluble TREM2 (sTREM2) activates the Akt-GSK3β signaling pathway^[35]^, upregulating GSK-3β activity and subsequently driving tau hyperphosphorylation^[36]^. This pathological phosphorylation disrupts microtubule dynamics through multiple mechanisms, which include the following. (1) Microtubule destabilization: hyperphosphorylated tau promotes the dissociation of normal microtubule-associated proteins from microtubules, compromising structural integrity and causing proteinaceous deposits that damage neurons^[37]^. (2) Subcellular trafficking defects: mislocalization of tau from axons to dendritic compartments disrupts synaptic formation and plasticity^[38,39]^. Synaptic dysfunction: reduced synaptophysin expression impairs neurotransmission^[40]^, leading to cognitive deficits such as impaired learning and memory. These cascading effects may predispose patients to POD.

Machine learning algorithms use computers and various computational methods to analyze data. By learning and mining the potential connections existing in the data, they train an effective model to be applied in decision-making or prediction. There have not been machine learning models using sTREM2 for predicting POD.We show that the CatBoost model performed the the highest accuracy for predicting POD by ROC and PRC analyses. Calibration analysis comparing model-predicted and actual POD rates suggested good predictive accuracy. The online calculator could predict POD occurrence in the internal validation cohort.

This study had limitations. First, the ongoing PNDABLE study examines a prospective cohort, and the database should be regularly updated during follow-up in the future. Secondly, this was a single-center study, and multicenter studies are warranted for further verification. Thirdly, we only focused on the association of CSF sTREM2 with POD, without considering other factors linked to POD pathogenesis, which might be confounding factors.

## Conclusion

In summary, this prospective cohort study demonstrated that elevated CSF sTREM2 is a preoperative risk factor for POD, which is partially mediated by tau and ptau. The CatBoost model can accurately predict the occurrence of POD. Age≥80 plus sTREM2 ≥ 20 000 pg/ml could increase 3-year mortality in POD cases. These findings highlight the critical need for implementing early detection strategies and targeted interventions in high-risk patient populations, which may mitigate POD incidence and improve postoperative survival outcomes.

## Data Availability

The data that support the findings of this study are available in the PNDABLE database. According to relevant regulations, the data could not be shared but could be requested from the corresponding author.

## Research registration unique identifying number (UIN)

The PNDABLE study has received ethical approval from the Ethics Committee of Qingdao Municipal Hospital and is registered with the China Clinical Trial Registry (Registration number: ChiCTR2000033439, registration date: 06/07/2020).
